# Supplementary material for: Effects of Orange Peel Extract on Laccase Activity and Gene Expression in Trametes versicolor
Source: J Fungi (Basel). 2024 May 22;10(6):370. doi: 10.3390/jof10060370 (PMC11205045; doi:10.3390/jof10060370)
Supplement: Supplementary file 1 [file jof-10-00370-s001.zip › jof-2958520-supplementary.pdf]

Supplementary Materials Vandelook *et al.*

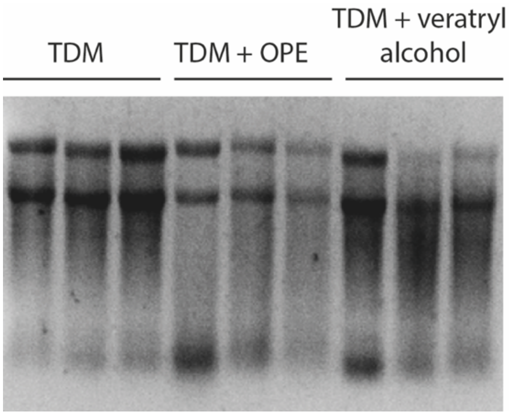

Supplementary Figure S1. Agarose gel electrophoresis of RNA samples extracted in 3 conditions (TDM = Trametes Defined Medium, OPE = orange peel extract)**.** For each condition, the results of 3 biological replicates are shown.

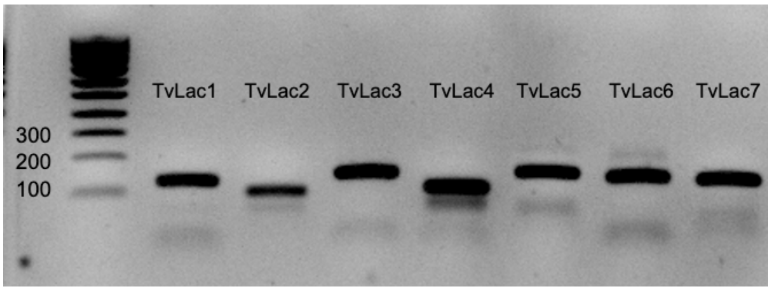

Supplementary Figure S2. Agarose gel electrophoresis of qPCR reaction products targeting amplification of each of the laccase genes using *Trametes versicolor* cDNA from the non-induced control group as a template. 0.1-1kbp DNA ladder.

```
TvLac1      MG----LQRF SFFVTLALVARSLAAIGPVASLVVANAPVSPDGFLRDAIVVNGVVPSPL
TvLac2      MTGLRLLPSFAALAVTVSLALNALAGIGPVLDLTISNAVVSPDGFSRAAVVANDQAPGPL
TvLac3      MSRFHSLLAF----VVASLTAVAHAGIGPVADLTITNAAVSPDGFSRQAVVVNGGTPGPL
TvLac4      MGRFSSLCAL---TAVIHSFGRVSAAIGPVTDLTISNADVSPDGFTRAAVLANGVFPGPL
TvLac5      MGKFHSFVNV--VALSLSLSGRVFGAIGPVTDLTISNADVTPDGITRAAVLAGGVFPGPL
TvLac6      M-SFSSLCRT---LVFLGACSSALASIGPVTELDIVNKVIAPDGVARDTVLAGGTFPGPL
TvLac7      M-SFRSLRRA---LVFLGVCGAAFAAIGPVTELDIVNKVIAPDGFARDTVLAGGTFPGPL
            *     :                 ..**** .* : *  ::***. * :::...  *.**

TvLac1      ITGKKGDRFQLNVVDTLTNHSMLKSTSIHWHGFFQAGTNWADGPAFVNQCPIASGHSFLY
TvLac2      ITGQMGDRFQINVVNKLSNHTMLKSTSIHWHGFFQKGTNWADGPAFVNQCPIATGHSFLY
TvLac3      ITGNMGDRFQLNVIDNLTNHTMLKSTSIHWHGFFQKGTNWADGPAFINQCPISSGHSFLY
TvLac4      ITGNKGDNFQINVIDNLSNETMLKSTSIHWHGFFQKGTNWADGAAFVNQCPIATGNSFLY
TvLac5      ITGNKGDEFQINVIDNLTNETMLKSTTIHWHGIFQAGTNWADGAAFVNQCPIATGNSFLY
TvLac6      ITGKKGDNFRINVVDKLVNQTMLTSTTIHWHGMFQHTTNWADGPAFVTQCPITTGDDFLY
TvLac7      ITGKKGDNFRINVVDKLVNETMLTATTIHWHGMFQHTTNWADGPAFVTQCPITTGHDFLY
            ***: **.*::**::.* *.:**.:*:*****:**  ******.**:.****::*..***

TvLac1      DFHVPDQAGTFWYHSHLSTQYCDGLRGPFVVYDPKDPHASRYDVDNESTVITLTDWYHTA
TvLac2      DFQVPDQAGTFWYHSHLSTQYCDGLRGPFVVYDPNDPHASLYDVDNDDTVITLADWYHTA
TvLac3      DFQVPDQAGTFWYHSHLSTQYCDGLRGPFVVYDPNDPAADLYDVDNDDTVITLVDWYHVA
TvLac4      DFTATDQAGTFWYHSHLSTQYCDGLRGPMVVYDPSDPHADLYDVDDETTIVTLSDWYHTA
TvLac5      DFTVPDQAGTFWYHSHLSTQYCDGLRGPLVVYDPDDPNASLYDVDDDTTVITLADWYHTA
TvLac6      NFRVPDQTGTYWYHSHLALQYCDGLRGPLVIYDPHDPQAYLYDVDDESTVITLADWYHTP
TvLac7      KFHVPDQTGTYWYHSHLSLQYCDGLRGPMVIYDPQDPQAHLYDVDDESTVITLADWYHTP
            .* ..**:**:******: *********:*:*** ** *  ****:: *::** ****..

TvLac1      ARLGPRFPLGADATLINGLGRSAS--TPTAALAVINVQHGKRYRFRLVSISCDPNYTFSI
TvLac2      AKLGPAFPPGSDATLINGLGRTAA--TPNADLAVISVTHGKRYRFRLVSMSCDPAYTFSI
TvLac3      AKLGPAFPLGADATLINGKGRSPS--TTTADLSVISVTPGKRYRFRLVSLSCDPNYTFSI
TvLac4      ASLGAAFPIGSDSTLINGLGRFAG--GDSTDLAVITVEQGKRYRMRLLSLSCDPNYVFSI
TvLac5      AKLGPAFPAGPDSVLINGLGRFSGDGGATNLTVITVTQGKRYRFRLVSISCDPNFTFSI
TvLac6      APLLP-----PAATLINGLGRWPG--NPTADLAVIEVQHGKRYRFRLVSTSCDPNYNFTI
TvLac7      APLIP-IPAMADSTLINGLGRWAG--NPTADLAVIEVEHGKRYRFRLVSISCDPNYNFTI
            * * .     . :.**** ** ..     : *:** *  *****:**:* **** : *:*

TvLac1      DGHNLTVIEVDGINSQPLLVDSIQIFAAQRYSFVLNANQTVGNYWVRANPNFGT-VGFAG
TvLac2      DDHSMTIIEADSVNTKPLEVDSIQIFAGQRYSFVLEANQDVGNYWVRADPLFGT-TGFDG
TvLac3      DGHNMTIIETDSINTAPLVVDSIQIFAAQRYSFVLEANQAVDNYWIRANPNFGN-VGFTG
TvLac4      DGHNMTIIEADAVNHEPLTVDSIQIYAGQRYSFVLTADQDIDNYFIRALPSAGT-TSFDG
TvLac5      DGHNMTIIEVDGVNHEALDVDSIQIFAGQRYSFILNANQSIDNYWIRAIPNTGT-TDTTG
TvLac6      DGHTMTIIEADGQNTQPHQVDGLQIFAAQRYSFVLNANQAVNNYWIRANPNRANTTGFAN
TvLac7      DGHSMTIIEADGENTQPLQVDKLQIFAAQRYSFVLHADQPVNNYWIRADPNLAH-TGFLQ
            *.*.:*:**.*. *  .  ** :**:*.*****:* *:* :.**::** *  .  ..
```

Supplementary Figure S3. Multiple sequence alignment of protein sequences of the 7 laccase isozymes (TvLac1 – TvLac7) in *Trametes versicolor*. Sequences originate from *T. versicolor* FP-101664 SS1 (accession numbers are given in Table 2 in the main manuscript). Copper-interacting residues present in the active site are indicated in yellow. "*" means identical amino acids, ":" indicates conserved substitutions, "." Indicates semi-conserved substitutions, *i.e.* amino acids with a similar shape. Continued on next page.

```
TvLac1      GINSAILRYQGAPVAEPTTTQTTSVIPLIETNLHPLARMPVPGSPTPGGVDKALNLAFNF
TvLac2      GINSAILRYDTASPTEPTTTQATSTKPLKETDLEPLASMPVPGSAVSGGVDKAINFAFSF
TvLac3      GINSAILRYDGAAAVEPTTTQTTSSAPLNEVNLHPLVATAVPGSPVAGGVDLAINMAFNF
TvLac4      GINSAILRYSGASEVDPTTTETTSVLPLDEANLVPLDSPAAPGDPNIGGVDYALNLDFNF
TvLac5      GVNSAILRYDTAEEIEPTTNATTSVIPLTETDLVPLDNPAAPGDPQVGGVDLAMSLDFSF
TvLac6      GINSAILRYKGAPIKEPTTNQTTIRNFLKETDLHPLTDPRAPGLPFKGGVDHALNLNLTF
TvLac7      GINSAILRYKGAPITEPTTNQTPSVKFLNEADLDPLTNPRAPGLPFRGGVDHAVNLKLTF
            *:*******. *   :***. :.    * *.:* **    .** .  **** *:.: :.*

TvLac1      NGTNFFINNASFTPPTVPVLLQILSGAQTAQDLLPAGSVYPLPAHSTIEITLPATAL---
TvLac2      NGSNFFINGATFQPPTTPVLLQIMSGAQAASDLLPSGDVYALPSDSTIELSFPATTG---
TvLac3      NGTNFFINGASFTPPTVPVLLQIISGAQNAQDLLPSGSVYSLPSNADIEISFPATAA---
TvLac4      DGTNFFINDVSFVSPTVPVLLQILSGTTSAADLLPSGSLFALPSNSTIEISFPITAT---
TvLac5      NGSNFFINNETFVPPTVPVLLQILSGAQDAASLLPNGSVYTLPSNSTIEISFPIITTDGA
TvLac6      NGSEFFINDAPFVPPTVPVLLQILNGTLDANQLLPPGSVYNLPPNSTIELSIPGG-----
TvLac7      NDSEFFINGAAFKPPTVPVLLQILNGTLDAHDLLPKGSVYSLPPYSTIELSIPGG-----
            :.::****. .* .**.******:.*:  * .*** *.:: **. : **:::*

TvLac1      --APGAPHPFHLHGHAFAVVRSAGSTTYNYNDPIFRDVVSTGTPAAGDNVTIRFQTDNPG
TvLac2      --APGAPHPFHLHGHTFAVVRSAGSTEYNYDNPIWRDVVSTGTPAAGDNVTIRFRTDNPG
TvLac3      --APGAPHPFHLHGHAFAVVRSAGSTVYNYDNPIFRDVVSTGTPAAGDNVTIRFRTDNPG
TvLac4      -NAPGAPHPFHLHGHTFSIVRTAGSTDTNFVNPVRRDVVNTGT--AGDNVTIRFTTDNPG
TvLac5      LNAPGAPHPFHLHGHTFSVVRSAGSSTFNYANPVRRDTVSTGN--SGDNVTIRFTTDNPG
TvLac6      --VTGGPHPFHLHGHAFSVVRSAGSTEYNYANPVKRDTVSIGL--GGDNVTVRFVTDNPG
TvLac7      --VRGGPHPFHLHGHTFSVVRSAGSDHYNYDNPVRRDTVSIGE--MGDNVTVRFVTDNPG
              . *.*********:*::**:***   *: :*: **.*. *    *****:** *****

TvLac1      PWFLHCHIDFHLEAGFAIVFAEDVADVKAANPVPKAWSDLCPIYDGLSEADQ
TvLac2      PWFLHCHIDFHLEAGFAVVMAEDIPDTKADNPVPQAWSDLCPIYDALDADDQ
TvLac3      PWFLHCHIDFHLEAGFAVVFAEDIPDVASANPVPQAWSDLCPTYDALDPSDQ
TvLac4      PWFLHCHIDFHLEAGFAIVFSEDTADVSNTTTPSTAWEDLCPTYNALDSSDL
TvLac5      PWFLHCHIDFHLDAGFAIVFAEDTADTASANPVPTAWSDLCPTYDALDSSDL
TvLac6      PWFLHCHIDFHLQAGLAIVFAEDAQDTKLVNPVPEDWNKLCPTFDKAMNITV
TvLac7      PWFLHCHIDFHLEAGLAIVFAEDTKATALVNPVPEEWEKLCPTYNASIHTNV
            ************:**:*:*::**   .   .. .  *..*** ::
```

Supplementary Figure S3. Continued.

A

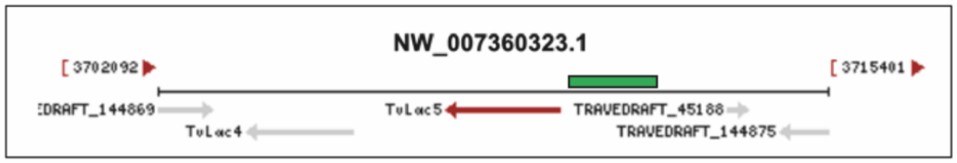

B

```
GTTGAAAACGAAAGCGGAGGCTGGGCGGCTAGAGAATGCGCTACGAGAAGACTGAAACGGGTGTCGTG
TGGGATGAGGATCGACGCTCTGAAGCTGAGGGATTTATACCTATAGCAAACGAGAGCGCCTGGGGCGT
ATGGAGGCGCGGCTTAGGATAGCTGCCAGACGTCAAAAATAGCGCGGACCGCTGCATCACCTAACTGC
GACGCTTTCAGTGCCGACGCGCAGCGCCGTCTCCCAAAGAATAGAACTCACCCTGCACAAATGTCTCG
CGGGTGTCGGGCACTAACACTGGTTCGGGTTCTGGTCGCTCGCCGCCGCCGGTTCGCAGGCGGGAGAA
GCGCGAGCACCGCCGTTCCGCAGACAGCACTTCACCAGCACAGATACCCGCTTGTAGACACCGGGTTT
GTGGCGACATATCCAAGGGATGGTTGCTGGTGGCGAGACAGTTGCTGGTGCACGGCGTCCATCCCTAC
CTTTCCTGCAGGTTTGATAGAGTACGCTGCATTCGAGTTCGCCTCCCGAAGGGTCTGTCAGGTGTGCG
CAGTACCCATGCTAGAAGCGAAGAGTTATGAGCCTGAGAGCCAAGGAGCGATGTCAGTGACGTCCACG
GCGTTCAGCCAGCCTCCGTCGTCCGACTGTCCGCATCAAAAGACAGCTGCGGACAGGGTAAGCTCATA
GACTGTGTGCGGGTGGAATGATAGAATGCAAGAACCTTGAAGGCATCGCAGAGTCCGGACTCCAGACT
CCGAGGTGGTGGAAGTGATGGCGGCTGCGAAGAGAGTTCTATGACGGCATTGGTCTGCGCACGCAGTT
CACCGTAACGAGGCTGAGGGACATCCATTTTTTGTGTCTCCCTATGTCAAACATTTTCACAGCCTACG
AGATTTTTCAACCGCACAAACGATCCGGATTATGGGCGTATACGCGAGTCGGTGGTGGTAGCAGCAAA
CAGCAACGCGGGGTACCAGCGCTTCCGCGACGCGCTTGCGATCAATTGATCGGGACAGACGCCCACGG
TACCCGGTGCGCGACTTTTGCGCAACATGCTTTCAGGATGCACTGGTCAACGGCTATAACCTCATTCT
TACGGAGTCTTTTCAGTCCTGTACAACGGTGAAGTGCATTGGTTTACAGTCTAACACATTGAAACCCA
CCTCATGAGGTCACTTAGACGGAAAATCACTACCGGACATCTTGCTCAAGTCCTACTCACCCGAGATC
GAGTTGGATATCAATCTCGAGTGAACTTAGTGTCCGTGTACCAGCACTTACTGCATCGACGTGAGCAG
TTTGAACGATAACTATGATGCTTGCTGCCGTTCTCTCCACTAAGTGAGCGATTGGCTCACCGGGTTCA
GACGCAATTCTCGGGTGCTTTGACGTTCTTCTCTGCTGCGCCGGTGACAAAGTACCGCGTTCTCTCTG
AACGGCATCAGCTGCTACAAGTGACGTGTACATTATCCCCGTTCGCCTGTAAGCCGCAATTATTGATA
TCTTACACCTCCGCTTGCTCATCTGGTACCGATGTCTAGATCAACGGGCATCTCGACATCCATTCCCC
GGCGGCAGCGATGGTCGGCTTTTTCCCAGGACCCGCAGCCGGGCACCCGCGGTCTATCGGAGGCTCAC
TGGATTATGGCTCAAGACGTCGTAGTGGGCCGCTCATGCTCTCCTTGGACCTCACCTCGCCGACGCGC
CCCATCTCTGGCATGACGCCGCTTTTGTTCTGCACCATACATGTCTCACTACGTGCGGAACCCAAACC
ACACTAGGACACGCGCGCCGTAGGGAGAACGTCGCCGCAGGCACAGTTCGGCACGCTCCTCGGATCGG
ACGCGGTTTTATAGTGGCGCAGGGCGGGATGGGAATGCTCGGCATTGAGCAGAGGCGCGTAGCCGTTT
GGCTTTGAGCCATCGTTTCACTGAAAGTTCGCGTCACGGTGGAGGAACGCTATACAGCGCAGTCTCCA
GCGCAAAGCTTATAACGCCTCATCCTGC
```

Supplementary Figure S4. Prediction of putative promoter and regulatory elements in the 2000-bp promoter region upstream of the CDS of TvLac5. **A**. Schematic overview of the genomic environment of the TvLac5-encoding gene in *T. versicolor*. The considered promoter region is indicated with a green bar. **B**. Promoter sequence with indication of TATA box in yellow (-101/-107), putative metal-responsive elements in green with consensus TGCRCNC (-540/-546 and -804/-809) and putative CreA binding sites in red with consensus SYGGRG (-13/-19, -128/-133, -740/-745, -960/-965, -1327/-1332, -1505/-1510, -1561/-1566, -1943/-1948, -1968/-1973). Position numbering is with respect to CDS start of the TvLac5-encoding gene. Please note that the orientation of the sequence is 5′ -> 3′ top strand with respect to the orientation of the schematic overview in A, and not with respect to the TvLac5 gene orientation.
